# Identification of the Genome Segments of Bluetongue Virus Serotype 26 (Isolate KUW2010/02) that Restrict Replication in a *Culicoides sonorensis* Cell Line (KC Cells)

**DOI:** 10.1371/journal.pone.0149709

**Published:** 2016-02-18

**Authors:** Gillian D. Pullinger, Marc Guimerà Busquets, Kyriaki Nomikou, Mark Boyce, Houssam Attoui, Peter P. Mertens

**Affiliations:** Vector-borne Viral Diseases Programme, The Pirbright Institute, Pirbright, Woking, Surrey, United Kingdom, GU24 0NF; Rega Institute for Medical Research, BELGIUM

## Abstract

Bluetongue virus (BTV) can infect most ruminant species and is usually transmitted by adult, vector-competent biting midges (*Culicoides* spp.). Infection with BTV can cause severe clinical signs and can be fatal, particularly in naïve sheep and some deer species. Although 24 distinct BTV serotypes were recognized for several decades, additional ‘types’ have recently been identified, including BTV-25 (from Switzerland), BTV-26 (from Kuwait) and BTV-27 from France (Corsica). Although BTV-25 has failed to grow in either insect or mammalian cell cultures, BTV-26 (isolate KUW2010/02), which can be transmitted horizontally between goats in the absence of vector insects, does not replicate in a *Culicoides sonorensis* cell line (KC cells) but can be propagated in mammalian cells (BSR cells). The BTV genome consists of ten segments of linear dsRNA. Mono-reassortant viruses were generated by reverse-genetics, each one containing a single BTV-26 genome segment in a BTV-1 genetic-background. However, attempts to recover a mono-reassortant containing genome-segment 2 (Seg-2) of BTV-26 (encoding VP2), were unsuccessful but a triple-reassortant was successfully generated containing Seg-2, Seg-6 and Seg-7 (encoding VP5 and VP7 respectively) of BTV-26. Reassortants were recovered and most replicated well in mammalian cells (BSR cells). However, mono-reassortants containing Seg-1 or Seg-3 of BTV-26 (encoding VP1, or VP3 respectively) and the triple reassortant failed to replicate, while a mono-reassortant containing Seg-7 of BTV-26 only replicated slowly in KC cells.

## Introduction

Arthropod-borne viruses (arboviruses) are transmitted between their vertebrate hosts (e.g. mammals or birds) by hematophagous arthropod vectors, especially mosquitos, midges or ticks. Unlike other viruses, they require the ability to complete their replication cycle in two disparate host-species. The arboviruses are predominately RNA viruses [1] belonging to the families *Flaviviridae*, *Togaviridae*, *Bunyaviridae*, *Rhabdoviridae*, and *Reoviridae*, along with a single DNA virus (African swine fever virus) belonging to the family *Asfarviridae* [1].

*Bluetongue virus* is the ‘type species’ of the genus *Orbivirus* within the family *Reoviridae* [2]. The bluetongue virus (BTV) can infect all ruminant species, as well as camelids and occasionally large carnivores [3–5]. Clinical signs of bluetongue disease (BT) are more severe in naïve animals, and are most commonly observed in sheep, and in white-tailed deer (e.g. in North America), although they are also seen (less frequently) in cattle and other species [6, 7]. The normal route of BTV transmission is via adults of vector-competent species of biting midge (*Culicoides* spp.), in which the virus also replicates. In addition, BTV can be transmitted via an oral route, or by vertical transmission in its ruminant hosts [8, 9]. Orbiviruses usually establish persistent infections in their vectors with no deleterious effects, and phylogenetic analyses indicate that they have evolved by co-speciation with their arthropod vectors [10]. Although *Culicoides* are regarded as the major vector for BTV, the virus can also replicate in cells of other arthropods including mosquitoes, drosophila and ticks [11–14].

BTV particles are composed of three concentric protein shells, surrounding a genome composed of 10 linear segments of double-stranded (ds) RNA [15, 16]. The genome segments range in size from 3954 to 822 bp, and are identified as segment 1 to 10 (Seg-1 to Seg-10) in order of decreasing molecular weight [2]. The BTV genome codes for 7 virus-structural proteins (VP1 to VP7) and 5 distinct non-structural (NS) proteins (NS1, NS2, NS3/NS3a, NS4 and S10-ORF2) [17–19].

Sequencing and phylogenetic comparisons show that Seg-2, and to a lesser extent Seg-6, are the most variable components of the BTV genome (encoding BTV VP2—‘outer-capsid protein 1’: and VP5—outer-capsid protein 2, respectively). The sequences of BTV Seg-2 divide into distinct clades that correlate with the virus serotype, and can be used to ‘type’ novel isolates by sequencing and/or type-specific RT-PCR assays [20–22]. The sequences of Seg-2 from different BTV serotypes can be grouped into ‘nucleotypes’ (nucleotypes A to L), which also reflect the serological relatedness / cross-reactions between different serotypes [20, 22, 23].

Structural proteins VP3 and VP7 (encoded by Seg-3 and Seg-7), form the sub-core and core-surface layers of the BTV particle respectively. These proteins are more highly conserved between serotypes than the outer-capsid proteins [2, 8, 21, 22, 24–26]. The core surface protein VP7 has been identified as an immuno-dominant *Orbivirus* species / serogroup specific antigen and is therefore targeted by most serological diagnostic assays to detect BTV [27]. Earlier phylogenetic studies have shown that the conservation of Seg-3 sequences, allows them to be used to identify the members of individual *Orbivirus* species [28, 29].

BTV also encodes three minor enzymatic proteins, which are also highly conserved and are assembled within the central space of the sub-core particle. These include the RNA-dependent RNA polymerase—VP1; the capping enzyme -VP4; and the putative helicase VP6, encoded by Seg-1, Seg-4 and Seg-9 respectively [30].

Five non-structural proteins have been identified in BTV infected cells (the tubule protein—NS1; the viral inclusion body matrix protein—NS2; the virus-release protein—NS3/NS3a; and two recently discovered protein NS4 and S10-ORF2) [15, 17, 19, 31, 32]. These NS proteins are highly conserved across different BTV strains and serotypes [33, 34], although NS3/NS3a (encoded by Seg-10) can be more variable in other *Orbivirus* species, and represents the second most variable protein (after VP2) of AHSV [35, 36].

All of the BTV genome segments show significant nucleotide-sequence variations that at least partially correlate with the geographic origins of the virus. This suggests that the initial emergence of individual BTV serotypes was followed by a significant period of geographic isolation, allowing mutations to accumulate and generating geographically distinct virus lineages or ‘topotypes’ [20–22, 37].

Since 1998, multiple BTV types have emerged within Europe, events that have been linked to increased international trade, movement of insects and/or climate change in the region. This raised concerns about future threats posed by bluetongue and other related *Culicoides* transmitted diseases [8, 9, 38, 39]. During early 2008, an atypical BTV was detected in apparently healthy goats from the Toggenburg region of north eastern Switzerland [SWI/2008/01] [40]. Sequence analyses identified a novel BTV ‘type’ and showed that it did not belong to the ‘major’ eastern or western BTV topotypes previously identified [22]. Infection was experimentally confirmed in goats and the virus caused mild bluetongue-like disease in sheep, although subsequent attempts to propagate the virus in mammalian or insect cell cultures were unsuccessful [41, 42].

In February 2010, another orbivirus was successful isolated from sheep in Kuwait exhibiting clinical signs consistent with BT, by injection of washed blood into embryonated chicken eggs followed by passage onto BHK-21 cells (virus isolate KUW2010/02) [43]. Genomic dsRNA extracted from infected cells, generated an electrophoretic migration pattern in agarose-gels, typical for a BTV isolate. Identification of KUW2010/02 as BTV was confirmed by indirect antigen-sandwich ELISA, targeting the outer-core protein VP7. Whole genome sequence analysis of KUW2010/02 revealed 98% amino acid (aa) identity with BTV-25 in VP7, indicating a common ancestry, but only 81.2% identity in Seg-7, suggesting that these two viruses had diverged a long time ago and indicating high conservation-pressures on the aa sequence, structure and function of VP7 [44]. Comparisons of Seg-2 and Seg-6 sequences with those of other BTV serotypes, identified KUW2010/02 as a distinct 26^th^ BTV ‘type’, within a 12th Seg-2 nucleotype (L) and a 9th Seg-6 nucleotype (I) [44]. Virus neutralization tests (VNT), using antisera against the existing 25 BTV types failed to neutralise KUW2010/02, confirming its identity as BTV-26 [43]. However, phylogenetic analyses showed a high level of divergence in most of the conserved genome-segments between most BTV strains and BTV-26 (KUW2010/02) and/or BTV-25 [SWI/2008/01], placing them as representatives of two novel and distinct BTV topotypes [44]. In addition, a novel BTV strain has recently been identified that represents a putative BTV serotype 27 [45].

The kinetics of BTV-26 infection in sheep and goats are similar to those of BTV-25. However, the virus can be horizontally transmitted to uninfected, in-contact goats, which subsequently seroconvert [46, 47]. This indicated that unlike BTV-25, BTV-26 can be transmitted horizontally by direct contact and replicates in mammalian cells (BHK-21, BSR and Vero cells) *in vitro*, but does not replicate in KC cells.

Reverse genetics was used to generate mono-reassortants between BTV-26 and the reference strain of BTV-1 (Western topotype—which replicates well in both mammalian and insect cell cultures) (see Results). The time course of replication was assessed for each reassortant strain in mammalian and insect vector cell lines, to identify individual genome segments of BTV-26 that restrict the ability of the virus to replicate in *Culicoides* cells.

## Materials and Methods

### Virus propagation and cells

The parental virus strains used were obtained from the dsRNA virus collection at IAH Pirbright (see: www.reoviridae.org/dsRNA_virus_proteins/ReoID/BTV-isolates.htm). BTV-26 [KUW2010/02] was originally isolated from the blood of a sheep from Kuwait as previously described [43]. BTV-1 [RSArrrr/01] is the reference strain of BTV serotype 1, originally from South Africa.

Viruses were routinely propagated in BHK-21 cells (Baby hamster kidney, clone 13), obtained from the European Collection of Animal cell Cultures (ECACC– 84100501) in Glasgow MEM medium (Invitrogen) with 10% heat-inactivated foetal calf serum (FCS). BSR cells, a clone of BHK-21 cells [48], were grown in Dulbecco’s modified Eagle medium (DMEM) containing 5% FCS. KC cells, derived from *C*. *sonorensis* midges [49], were grown in Schneider’s medium containing 10% FCS with penicillin (100 U/ml) /streptomycin (100 μg/ml) and fungizone (2 μg/ml). *Aedes albopictus* (mosquito) C6/36 cells [50] were grown in Leibovitz’s L15 medium (PAA) with 10% FCS, 10% tryptose phosphate broth solution (Sigma T8159), and penicillin/streptomycin. Mammalian cells were incubated at 37°C in air with 5% CO_2_, and insect cells were incubated at 28°C.

### Preparation of viral dsRNA

Viral dsRNA was extracted from virus-infected cell cultures (grown in T175 flasks) showing advanced cytopathic effect (CPE) using Trizol reagent (Gibco BRL) [51]. Briefly, dsRNA was separated from contaminating ssRNA by precipitating in 2M lithium chloride (LiCl). Then dsRNA in the supernatant was precipitated by addition of three volumes of 100% ethanol and 0.25 volumes of 7.5M ammonium acetate (final ammonium acetate concentration of 0.44 M), washed twice with 70% ethanol and re-suspended in RNase-free water. In addition, viral RNA was purified from small volumes of cell culture medium using a Kingfisher robot (Thermo Scientific) with a MagVet Universal Isolation Kit (Laboratoire Service International).

### Full-length amplification of cDNAs (FLAC)

Full length cDNA copies of BTV genome segments were synthesised and amplified, after ‘anchor spacer–ligation’ as described previously [52].

### Preparation of cDNA clones and DNA sequence determination

Complete genome segments were amplified from the cDNA of BTV-1 RSArrrr/01 and BTV-26 KUW2010/02 by PCR using the primers shown in Table 1. The upstream primers contained a T7 promoter sequence and a restriction site for unidirectional cloning, while the downstream primers contained restriction sites for cloning and for linearization at the 3’ end of the segment, as described [53]. PCR was carried out using a PCR extender system (5 Prime) and the PCR products were double digested with the appropriate restriction enzymes. These were ligated into pGEX-4T-2, digested with the same enzymes, and transformed into competent cells of *E*. *coli* STBL2 (Invitrogen). Colonies obtained on ampicillin-containing agarose plates were confirmed by plasmid purification (Qiagen) and DNA sequencing (using the BigDye Terminator v3.1 Cycle Sequencing Kit, Invitrogen). Alternatively, plasmid clones for some segments were synthesized by GeneArt (see results section for details).

**Table 1:** Sequences of cloning primers.

Serotype, segment	Sequence (5’-3’)
**BTV-1 [RSArrr/01]**	
Seg-1 forward	TCTAGCGGATCCTAATACGACTCACTATAGTTAAAATGCAATGGTCGCAATC
Seg-1 reverse	TACAGTAAGCGGCCGCGTCTCAGTAAGTGTAATGCGGCGCGTGC
Seg-2 forward	TCTAGCGAATTCTAATACGACTCACTATAGTTAAAATAGTAGCGCGAT
Seg-2 reverse	TACAGTAAGCGGCCGCGTCTCAGTAAGTCTAATAGTGCGCGGATC
Seg-3 forward	TCTAGCGAATTCTAATACGACTCACTATAGTTAAATTTCCGTAGCCATGGCTG
Seg-3 reverse	TACAGTAAGCGGCCGCGTCTCAGTAAGTGTGTTCCCGCTGCCGC
Seg-4 forward	TCTAGCGAATTCTAATACGACTCACTATAGTTAAAACATGCCTGAGCCACACG
Seg-4 reverse	CGTAAGCGGCCGCGGTCTCAGTAAGTTGTACATGCCCCCCTC
Seg-5 forward	TCTAGCGAATTCTAATACGACTCACTATAGTTAAAAAAGTTCTCTAGTTGGC
Seg-5 reverse	TACAGTAAGCGGCCGCGTCTCAGTAAGTTGAAAAGTTCTAGTAGAGTG
Seg-6 forward	TCTAGCGAATTCTAATACGACTCACTATAGTTAAAAAGTGCGCCCTTAGCGAA
Seg-6 reverse	TACAGTAAGCGGCCGCGGTCTCAGTAAGTGTAAGTGCTTCCCGTCGC
Seg-7 forward	TCTAGCGAATTCTAATACGACTCACTATAGTTAAAAATCTATAGAGATGGACA
Seg-7 reverse	TACAGTAAGCGGCCGCGGTCTCAGTAAGTGTAATCTAAGAGACGTTTG
Seg-8 forward	TCTAGCGGATCCTAATACGACTCACTATAGTTAAAAAATCCTTGAGTCATGGAG
Seg-8 reverse	TACAGTAAGCGGCCGCGTCTCAGTAAGTGTAAAATCCCCCCCTAACC
Seg-9 forward	TCTAGCGAATTCTAATACGACTCACTATAGTTAAAAAATCGCATATGTCAGCTG
Seg-9 reverse	TACAGTAAGCGGCCGCGTCTCAGTAAGTGTAAAATCGCCCTACGTCA
Seg-10 forward	TCTAGCGAATTCTAATACGACTCACTATAGTTAAAAAGTGTCGCTGCCATGCT
Seg-10 reverse	TACAGTAAGCGGCCGCGTCTCAGTAAGTGTGTAGCGCCGCATACCCTC
**BTV-26 [KUW2010/02]**	
Seg-2 forward	GTGATGGATCCTAATACGACTCACTATAGTTAAAAGAGCGTTCCACCAT
Seg-2 reverse	GGTATGCGGCCGCGTCTCAGTAAGTGTAAGAGGCCACCGG
Seg-4 forward	GTGATGGATCCTAATACGACTCACTATAGTTAAAACATGCCTGAGCCAC
Seg-4 reverse	CAGTAAGCGGCCGCGGTCTCAGTAAGTTGTAACATGCCCCC
Seg-5 forward	GTGATGGATCCTAATACGACTCACTATAGTTAAAAAAGTTCTCTAGTCG
Seg-5 reverse	CAGTAAGCGGCCGCCTCTTCAGTAAGTTGAAAAGTTCTATTAGAG
Seg-6 forward	GTGATGGATCCTAATACGACTCACTATAGTTAAAAAGTACCCTCTAACTCG
Seg-6 reverse	GTATGCGGCCGCGTCTCAGTAAGTGTAAGCACCTCCCCC
Seg-7 forward	GTGATGGATCCTAATACGACTCACTATAGTTAAAAATCTATAGAGATGGACACT
Seg-7 reverse	GGTATGCGGCCGCGAAGACCAGTAAGTGTAATCTAAGAGACG
Seg-8 forward	GTGATGGATCCTAATACGACTCACTATAGTTAAAAAATCCTTAGTCATGG
Seg-8 reverse	GTATGCGGCCGCGTCTCAGTAAGTGTAAAATCCCCCCCT
Seg-9 forward	GTGATGGATCCTAATACGACTCACTATAGTTAAAAAATCGCTTATGTCGG
Seg-9 reverse	CAGTAAGCGGCCGCGGTCTCAGTAAGTGTAAAACCGCTATATG
Seg-10 forward	GTGATGGATCCTAATACGACTCACTATAGTTAAAAAGTGTCGCTGCCAT
Seg-10 reverse	GGTATGAATTCGTCTCAGTAAGTGTGTAGTGCCGCATA

### Reverse genetics and confirmation of recombinant viruses

Capped positive sense transcripts were generated by linearization of plasmid DNA from each clone, by digestion with appropriate restriction enzymes, followed by *in vitro* transcription using a mMessage mMachine T7 Ultra Kit (Life Technologies) as described [53]. To rescue reassortant viruses, BSR cells at approximately 90% confluence were transfected with a mixture of the appropriate capped transcripts, in a two-step procedure [53]. First, transcripts from segments 1, 3, 4, 5, 8 and 9 (coding for VP1, VP3, VP4, NS1, NS2 and VP6) of the required reassortant were transfected (400 ng each in a 6 well format) using Lipofectamine 2000. After 18–20h, the cells were transfected with a mixture of RNAs containing all ten transcripts from the virus to be ‘rescued’ (400 ng of each). Transfected BSR cells were then incubated for 2–3 days, before passage onto fresh BSR cells (80–90% confluent) in T25 flasks as described previously [54]. These were examined daily for the appearance of CPE. For reassortants which failed to rescue in this way (those with segments 1–3 of BTV-26, see Results) attempts were made to modify the first transfection step. In these cases, expression plasmids, pCDNA3.1 containing open reading frames of Seg-1, 3, 4, 5, 8 and 9 from BTV-1 RSArrrr/01 were used in the first transfection step, instead of transcripts as described previously [55].

To confirm rescue of the required viruses, RNA was extracted from cultures showing advanced CPE using the Trizol method (see above), and cDNA was prepared from at least two genome segments of the rescued viruses (including the heterologous segment from monoreassortants), using a Superscript III One-Step RT-PCR with Platinum Taq kit (Invitrogen) according to the manufacturer’s instructions. Approximately 500–1000 bp regions were amplified by this method, and this was followed by DNA sequencing.

The identity of the rescued viruses was further verified by comparing migration of their purified viral dsRNA to the parental strains of BTV-1 and BTV-26, by electrophoresis on 11% SDS-PAGE gels [15].

### Plaque purification

A volume of 0.5 ml of the suitable virus dilutions, prepared in DMEM with 5% FCS, were added to confluent monolayers of BSR cells in 12 well plates. After 90 min at 37°C with 5% CO_2_, the diluted virus was removed and replaced with 3ml of MEM containing 3% FCS and 1.5% melted agarose type V11A (Sigma-Aldrich). Plates were incubated at 37°C with 5% CO_2_, until plaques were visible. These were picked into DMEM/5% FCS, before passage on BSR cells.

### Virus titrations

To determine the titre of stock viruses or supernatant samples from time-course experiments, DMEM (100μl) containing 1% FCS and penicillin / streptomycin was aliquotted into each well of 96 well plates. Virus (50μl) was added to the first well, then serially diluted across the plate to give half-log dilutions. Each virus was tested in 4 or 8 rows. The last well was a negative control with no virus. BSR cells in DMEM containing 5% FCS were then added to each well (3 x 10^5^ cells per well in 100μl). Plates were incubated for 6 days, and the number of wells with CPE was determined. TCID_50_ values were calculated using the Karber formula [56]. For calculation of multiplicity of infection (MOI), a PFU/ml working estimate was calculated using the formula, one TCID50 = 0.7 PFU/ml; [56]).

### Timecourse experiments

Timecourse experiments were carried out for parent and reassortant in different cell lines. T25 flasks were seeded with 1.5 x 10^6^ BSR cells and after 24h (at approximately 80% confluence) were infected at an MOI of 0.04. After 30 mins at 37°C 250 μl samples (T_0_ samples) of the medium were collected The infected cells were further incubated at 37°C and samples of medium (250 μl) were taken at time intervals up to 96 h (at 8h, 24h, 32h, 48h, 56h, 72h and 96h). Insect cells were infected in suspension after determining the cell number using a haemocytometer. Infections were carried out at an MOI of approximately 0.04 and incubated for 30 mins at room temperature. T_0_ samples were collected as for BSR cells and then the infected cells were transferred to T25 tissue culture flasks and incubated at 28°C. Samples of medium (250 microlitres) were collected at intervals up to 14 days post-infection.

The samples were stored at 4°C until the end of the experiment and then viral RNA was extracted from 100 μl volumes using a Kingfisher robot. Viral RNA was quantified using the segment 9-specific real-time RT-PCR assay [57] with the Superscript III One-Step qRT-PCR with Platinum Taq Kit (Invitrogen), and expressed as genome copies per reaction (each reaction contained RNA from 5 microlitres of tissue culture supernatant). To standardize the data, a dilution of BTV-1 [RSArrrr/01] viral RNA was included on every PCR plate. In one experiment in KC cells, infectious virus was also quantified in all samples by end-point titration on BSR cells to confirm that measurement of viral RNA replication gave comparable results to measurement of infectious virus, and was an appropriate method to quantify viral replication. The virus titers were expressed as tissue culture infective dose (TCID_50_)/ml.

## Results

### Generation of plasmid clones

Most of the genome segments from BTV-1 [RSArrrr/01] and BTV-26 [KUW2010/02] were successfully cloned as described in Material and Methods. The inserts were completely re-sequenced and those with the genome consensus sequence were selected where possible. A few clones had conservative changes in the coding regions (BTV-1 Seg-3, Seg-5 and Seg-8, and BTV-26 Seg-2 and Seg-8) but none had changes in the untranslated regions. Clones of Seg-1 and Seg-3 from BTV-26 were not obtained by this method. The relevant cDNA copies with upstream T7 promoters were therefore synthesized and subcloned (Gene Art) and plasmids containing the synthetic inserts were used directly for *in vitro* transcription.

### Generation of reassortant viruses using reverse genetics

Seven of the ten possible mono-reassortant viruses (containing a single BTV-26 genome-segment and nine BTV-1 segments) were obtained by the original procedure (see Materials and Methods). These reassortants contain segments 4 to 10 of BTV-26. Their identities were confirmed by partial DNA sequencing, and they are designated as BTV-1_26-S4_, etc, where the uppercase section of the name refers to the BTV-1 [RSArrrr/01] backbone, with subscript referring to the added BTV-26 segment. Attempts to rescue mono-reassortants containing Seg-1 or Seg-3 of BTV-26 (BTV-1_26-S1_ and BTV-1_26-S3_), using lipofection of RNA transcripts were unsuccessful. However, the required mono-reassortants were subsequently generated by use of ‘expression plasmids’ in the first transfection step. With both these reassortants, there was also a longer lag before CPE was visible (4–5 days after passage of the transfected wells) than for the other reassortants (approximately 2 days after passage).

Several attempts to rescue BTV-1_26-S2_ were unsuccessful, despite using either transcripts or expression plasmids for the first transfection step. It was considered possible that VP2 of BTV-26 was incompatible with proteins VP5 and VP7 of BTV-1 (encoded by Seg-6 and Seg-7), with which it would interact in the virus particle [58, 59]. A triple reassortant containing all three segments from BTV-26 (BTV-1_26-S2, S6, S7_) was successfully rescued, although attempts to generate double reassortants BTV-1_26-S2, S6_ or BTV-1_26-S2, S7_ were unsuccessful. Generated reassortants are summarized in Table 2.

**Table 2:** The genome-segment composition of reassortants generated between BTV-1 [RSArrrr/01] and BTV-26 [KUW2010/02].

Virus	Seg-1	Seg-2	Seg-3	Seg-4	Seg-5	Seg-6	Seg-7	Seg-8	Seg-9	Seg-10
BTV-1_26 S1_	26									
BTV-1_26 S2,S6,S7_		26				26	26			
BTV-1_26 S3_			26							
BTV-1_26 S4_				26						
BTV-1_26 S5_					26					
BTV-1_26 S6_						26				
BTV-1_26 S7_							26			
BTV-1_26 S8_								26		
BTV-1_26 S9_									26	
BTV-1_26 S10_										26

26 indicates those genome-segments derived from BTV-26. Other segments are from BTV-1.

In addition, BTV-1 [RSArrrr/01] virus was rescued by the original procedure, and this virus was used for all the subsequent analyses. However, despite many attempts, BTV-26 [KUW2010/02] has so far not been rescued, and the originally isolated virus was therefore used throughout.

**Fig 1:**
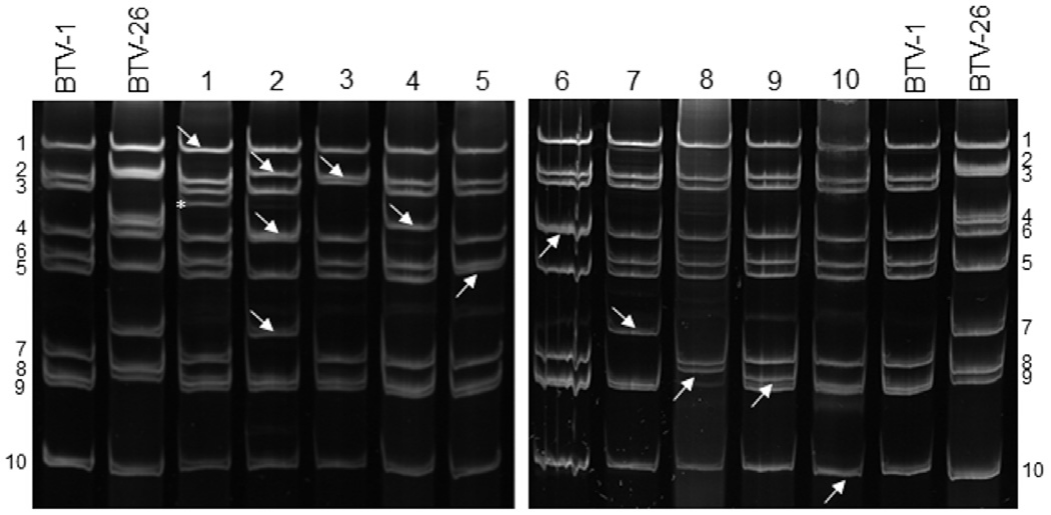
Viral RNA profiles of reassortant and parent viruses. Viral RNA segments were separated by 11% SDS-PAGE. Lane 1, BTV-1_26-S1_; 2, BTV-1_26-S2,S6,S7_; 3, BTV-1_26-S3_; 4, BTV-1_26-S4_; 5, BTV-1_26-S5_; 6, BTV-1_26-S6_; 7, BTV-1_26-S7_; 8, BTV-1_26-S8_; 9, BTV-1_26-S9_; and 10, BTV-1_26-S10_. The segments derived from BTV-26 are indicated by arrows. The asterisk shows an extra band observed in BTV-1_26-S1_ (see text).

Most of the genome segments from the two parent viruses (BTV-1 and BTV-26) migrate at different rates during SDS-PAGE, with the exception of Seg-1 which could not be clearly differentiated. The parental and reassortant viruses that were generated were verified by SDS-PAGE of their dsRNA segments (Fig 1). The reassortants all showed the expected migration patterns, with the exception of BTV-1_26-S1_ which had an extra band below the segment 3 band (indicated by an asterisk, Fig 1). This band was purified from an agarose gel and sequenced, which showed it was a truncated form of Seg-3. This virus was therefore plaque purified before further analysis (so that it contained only the full-length Seg-3—data not shown).

### Replication kinetics of reassortant BTV strains

**Fig 2:**
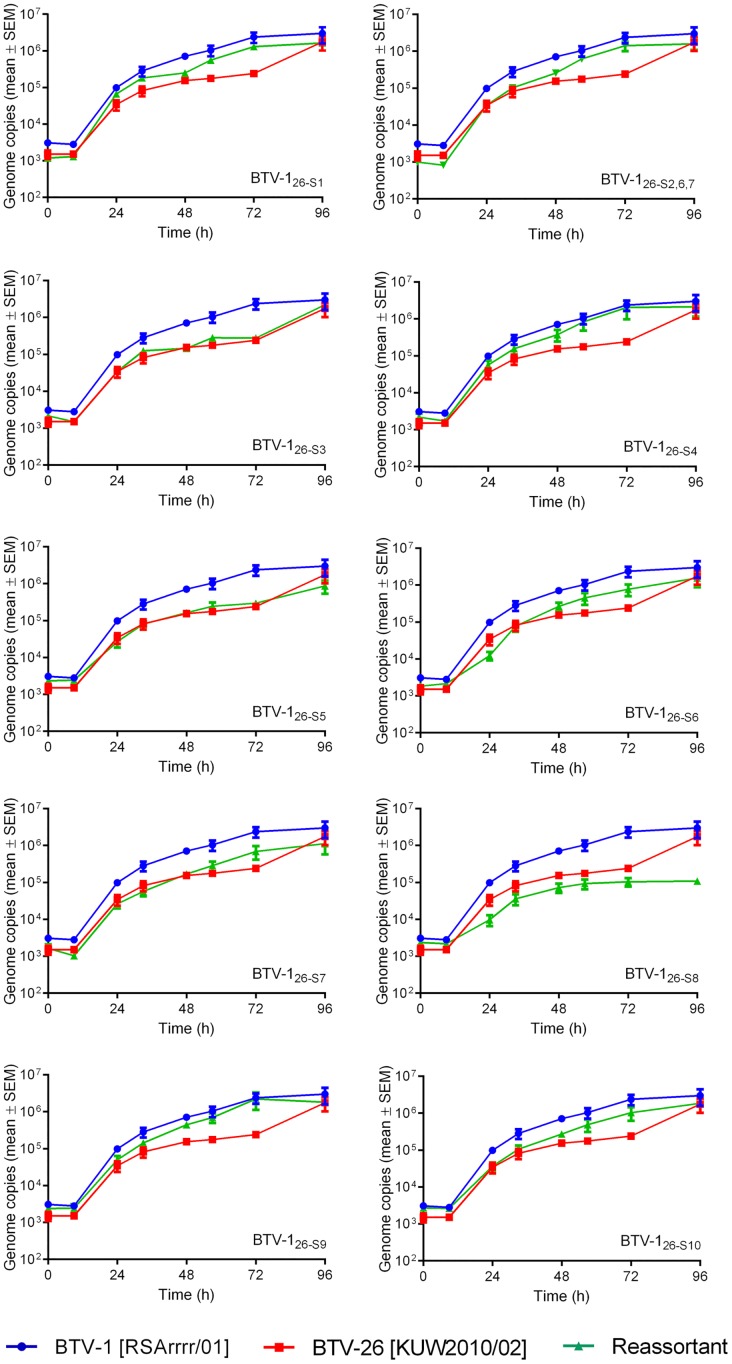
Viral RNA replication of parent and mono-reassortant viruses in BSR cells. Growth curves were generated as described in Materials and Methods. Each virus was tested in three separate time course experiments, and the amount of viral RNA in each 5 microlitre sample of tissue culture supernatant was measured by real-time RT-PCR in duplicate wells. Points represent the mean of the six values obtained for each sample. Error bars show the standard error of the mean.

In BSR cells, the parental strains of BTV-1 [RSArrrr/01] and BTV-26 [KUW2010/02] both replicated well, reaching equivalent final genome copy numbers per reaction, although BTV-26 was somewhat slower with lower RNA copies at 24 to 72 hours post infection (h.p.i., Fig 2). Most of the reassortants also replicated well in these mammalian cells, with growth kinetics intermediate between the two parent viruses, although mono-reassortants containing Seg-3, 5, 6 or 7 of BTV-26, matched the slower growth rate of BTV-26. Only reassortant strain BTV-1_26-S8_ (expressing NS2 of BTV-26) had a markedly slower replication rate, with a final genome copy number of over a log lower than either parental strain at 48 to 96 h.p.i.

**Fig 3:**
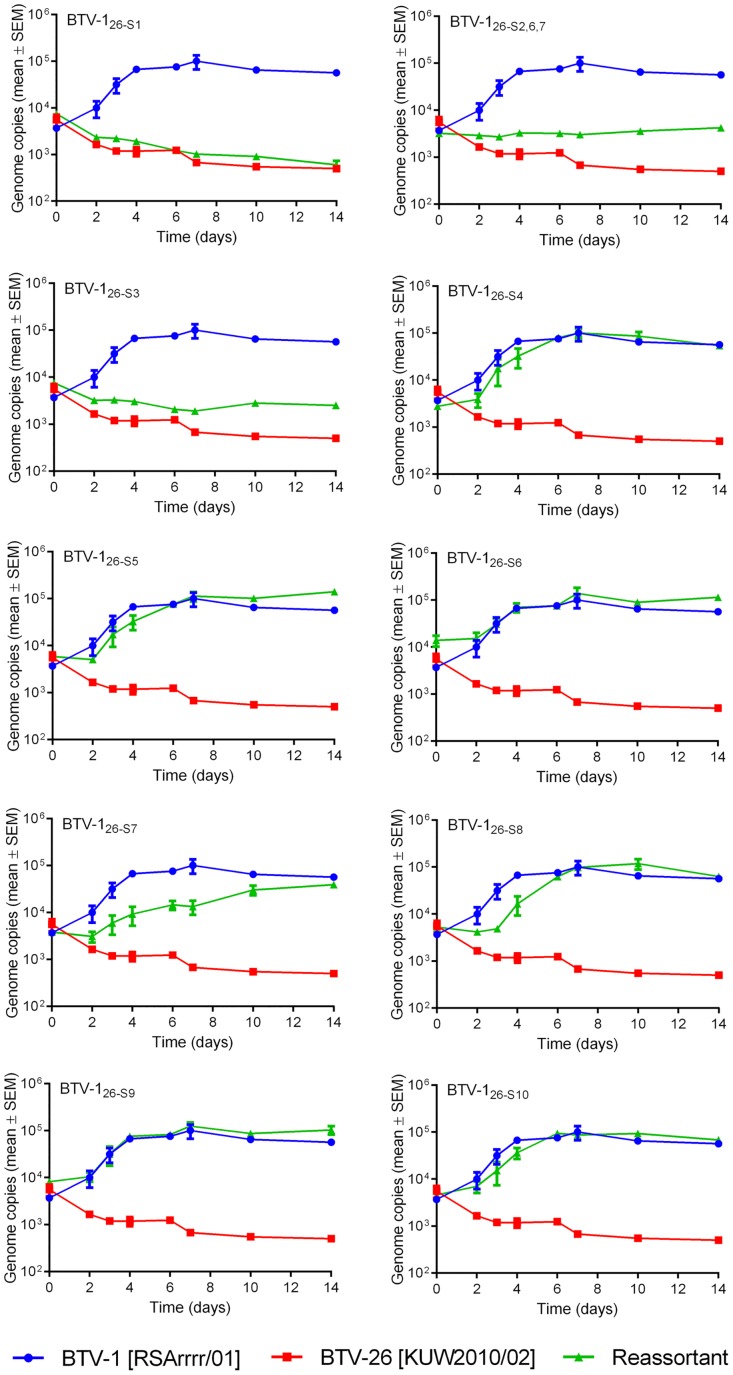
Viral RNA replication of parent and mono-reassortant BTV strains in KC cells. Growth curves were generated as described in Materials and Methods. Each virus was tested in three separate time course experiments, and viral RNA from each 5 microlitre sample of tissue culture supernatant was measured by real-time RT-PCR in duplicate wells. Points represent the mean of the six values obtained for each sample. Error bars show the standard error of the mean.

**Fig 4:**
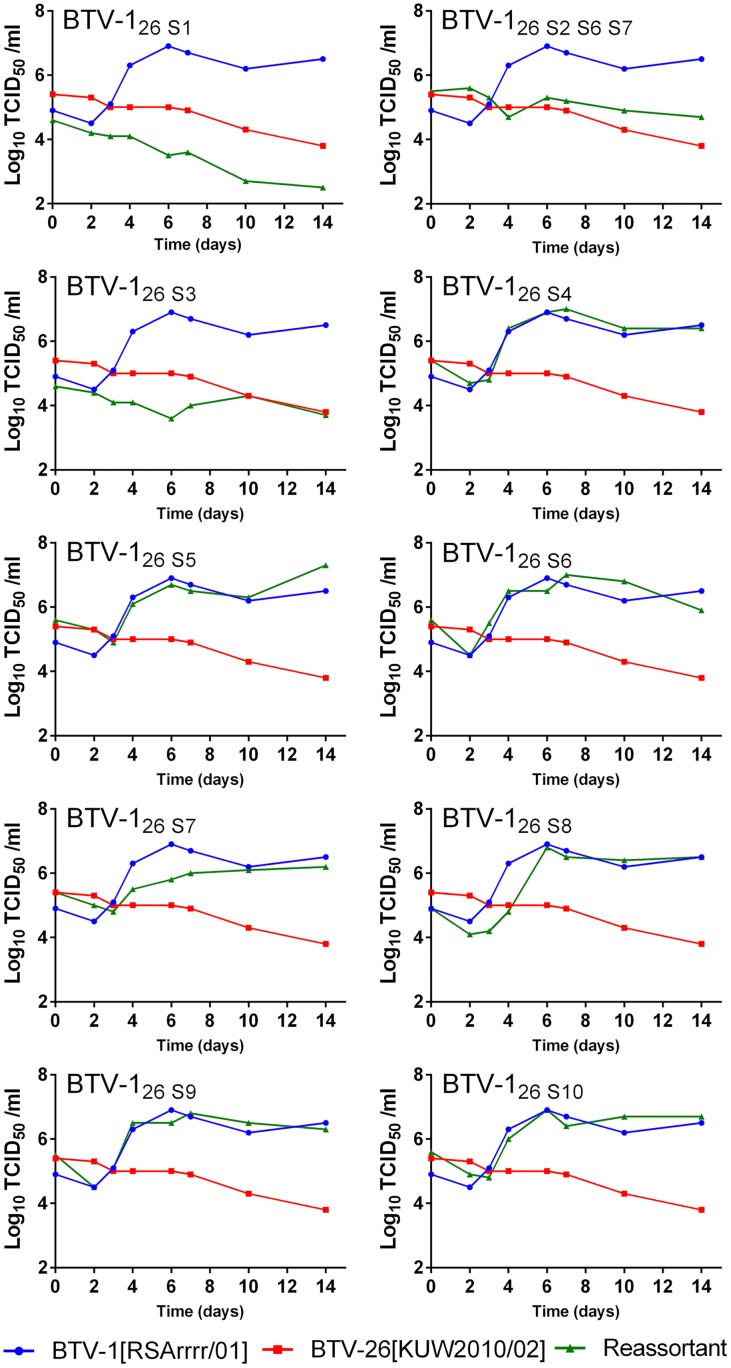
Viral growth kinetics of parent and mono-reassortant BTV strains in KC cells by endpoint titration. The results were from a single KC timecourse experiment. Infectious virus was measured from each sample by end-point titration, and results show the log_10_ TCID_50_/ml.

The BTV-26 parental strain failed to replicate in KC cells, showing a steady, gradual decline in genome copy number per reaction (Fig 3). In contrast the BTV-1 parental strain did replicate but achieved a genome copy number of only ~ 10^5^ per 5 microlitres of tissue culture supernatant (by 7 days post infection (d.p.i.)), compared with over 10^6^ in BSR cells (by 4 d.p.i) (Fig 3). Several of the reassortant strains replicated with similar kinetics to BTV-1 in KC cells, these included BTV-1_26-S4_, BTV-1_26-S5_, BTV-1_26-S6_, BTV-1_26-S9_ and BTV-1_26-S10_. However, there was an initial lag in the replication (RNA synthesis) of BTV-1_26-S8_ in KC cells (by approximately 3 days), which was not observed in BSR cells or with the parental BTV-1 strain, in either cell line. There was a significant delay (an eclipse period of at least 2 days, ~four days for BTV-1_26-S8_) in the rise in infectivity of all of the strains that replicated in KC cells, (including the BTV-1 parental strain), with the same final titre achieved by BTV-1 and BTV-1_26-S8_ at 6 d.p.i. in KC cells (Figs 3 and 4).

Four of the reassortant strains showed much reduced, or no replication in the *Culicoides* cells. BTV-1_26-S1_ failed to replicate, showing a similar gradual reduction in viral RNA copies to the BTV-26 parent strain, over 14 days (Fig 3). Titration results confirmed that virus infectivity also decreased throughout the incubation period (Fig 4). Reassortant BTV-1_26-S3_ and triple reassortant BTV-1_26-S2, S6, S7_ also failed to replicate productively in KC cells, with viral RNA levels and infectious virus titres that either showed a small decrease, or for strain BTV-1_26-S2, S6, S7_ were unchanging over the two week incubation period (Figs 3 and 4). In contrast, BTV-1_26-S7_ was able to replicate in KC cells, but it did so at a substantially reduced rate (as assessed by both viral RNA levels and infectious virus titre) compared to the BTV-1 parent strain. The infectivity of BTV-1_26-S7_ only increased by about 10 fold, in contrast to BTV-1 [RSArrrr/01] which increased by ~100 fold at its peak (Fig 4).

**Fig 5:**
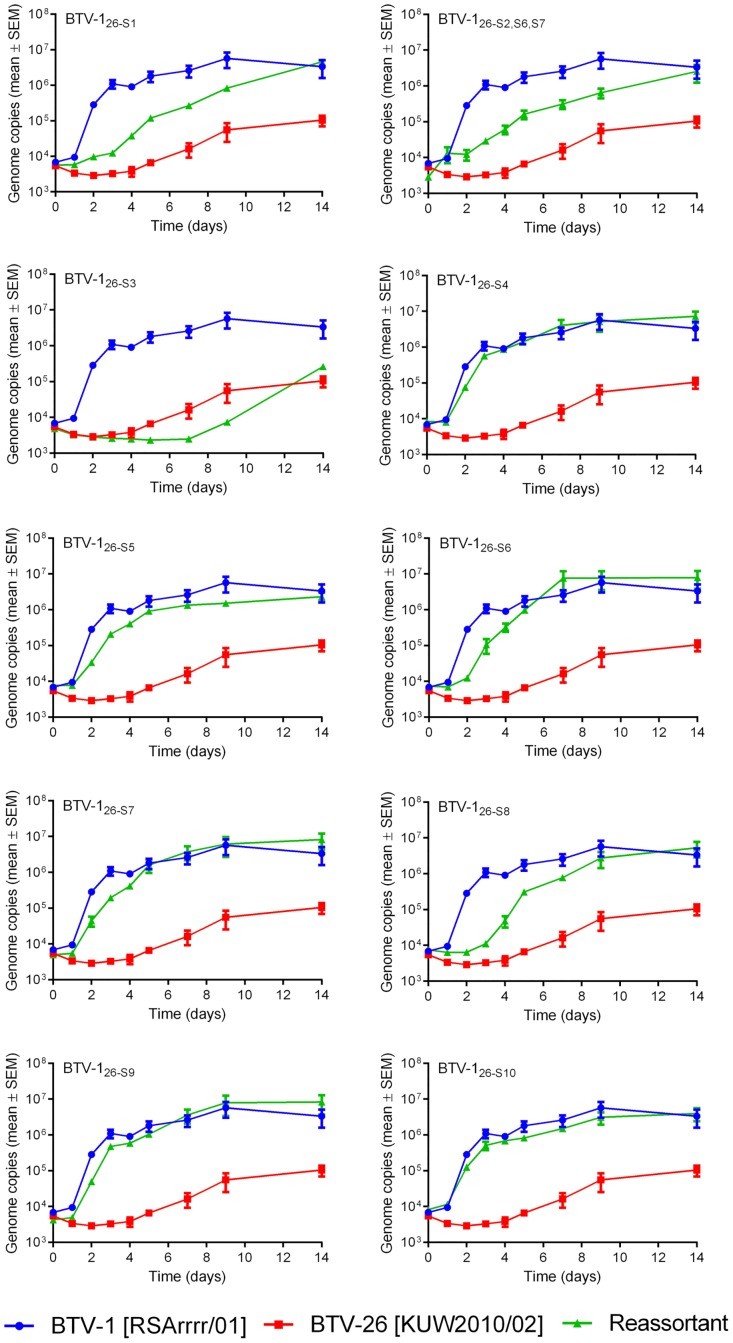
Viral RNA replication of parent and mono-reassortant viruses in C6/36 cells. Growth curves were derived as described in Materials and Methods. Each virus was tested in three separate time course experiments, and viral RNA from each 5 microlitre sample of tissue culture supernatant was measured by real-time RT-PCR in duplicate wells. Points represent the mean of the six values obtained for each sample. Error bars show the standard error of the mean.

A time course for BTV-1 replication in C6/36 cells (Fig 5) showed levels of viral RNA synthesis comparable to those achieved in BSR cells (Fig 2), generating considerably larger amounts of RNA than produced in KC cells (Fig 3). Although BTV-26 also replicated in C6/36 cells, it was significantly slower than BTV-1, with levels of viral RNA only starting to increase at 4 days post-infection, and reaching a lower final titre at 14 days post-infection (Fig 5). The levels of BTV-26 replication in mosquito cells are comparable to those achieved by BTV-1 in KC cells.

All of the reassortants also replicated in C6/36 cells (Fig 5). Those strains that showed no significant increase, or even a decrease in virus titre in KC cells (BTV-1_26-S1_; BTV-1_26-S2, S6, S7_ and particularly BTV-1_26-S3_) also replicated more slowly in C6/36 cells, although viral RNAs continued to rise up to 14 d.p.i. The other reassortants generated similar levels of viral RNA to BTV-1 by day 14, although with some initial delays. In particular BTV-1_26-S8_, which replicated to lower levels in BSR cells and showed a significant delay in KC cells, also showed a delay of ~3 days, during the early stages of replication in C6/36 cells. BTV-1_26-S7_, which showed a large delay in replication in KC cells, showed only a minor delay in replication in C6/36 cells.

## Discussion

BTV-26 [KUW2010/02] replicates in mammalian cells (BHK or BSR cells) *in vitro* but unlike BTV-1 [RSArrrr/01] and most other BTV strains that have been tested, does not replicate productively in KC cells, which are derived from the BTV-competent vector, *C*. *sonorensis*. A series of reassortant strains containing individual genome segments (or a selected combination of segments) from BTV-26, were generated within a BTV-1[RSArrrr/01] genetic ‘backbone’, making it possible to identify individual genome segments that restrict replication in KC cells.

Initial attempts to rescue Seg-2 of BTV-26, as a mono-reassortant within the BTV-1 backbone, were unsuccessful. However, this segment was recovered as a component of BTV-1_26- S2, S6, S7_, a triple reassortant that has both outer capsid proteins (VP2 and VP5) and the outer-core protein (VP7) from BTV-26. This suggests that physical interactions that are known to exist between VP2, VP5 and VP7 [58, 59] allow VP2 of BTV-26 to be incorporated and fully functional within the virus particle. BTV-1_26- S2, S6, S7_ replicates ‘normally’ in BSR cells, but failed to replicate in KC cells. Mono-reassortant BTV-1_26-S6_, replicated in KC cells with similar kinetics to the BTV-1 parental strain, while BTV-1_26-S7_ replicates substantially slower in KC cells than BTV-1. This indicates that both VP2 and VP7 of BTV-26 can either restrict or reduce infection and/or replication in KC cells.

Previous reverse genetics studies between the more closely related Western-topotype strains of BTV-1 and BTV-8, showed that all possible mono-reassortants could be rescued by reverse genetics [60–62]. Further investigations will be needed to determine the relative importance of the major BTV topotypes in reassortment compatibility groups. Our findings match studies of natural reassortants of a closely related orbivirus species, epizootic hemorrhagic disease virus (EHDV), showing that the genome segments encoding VP2, VP5 and VP7 from serotype 1 and serotype 2, preferentially remain within the homologous serotype during co-infections, forming a reassortment ‘linkage group’ [63]. This is further supported by evidence that VP2 and to a lesser extent VP5, both show sequence variations that correlate with BTV serotype. Interestingly, in a recent study, a double reassortant containing just the genome segments for VP2 and VP5 of BTV-26 in the BTV-1 backbone was successfully rescued [64].

A primary function of outer-capsid protein VP2 is in cell attachment during initiation of infection (51). Analyses of its replication time-course suggest that BTV-26 may be unable to bind to or initiate infection in KC cells. If the virus failed to enter KC cells, and consequently did not ‘uncoat’ during the early stages of infection, it would presumably remain intact in the tissue culture media, which may explain the stable RNA signal observed throughout the BTV-1_26- S2, S6, S7_ time course in KC cells.

Although the triple reassortant replicates slowly in C6/36 mosquito cells, it was faster than the BTV-26 parent, suggesting that VP2 of BTV-26 can mediate attachment to C6/36 cells but possibly less efficiently than to BSR cells. The incorporation of VP5 or VP7 of BTV-26 reduced the initial replication rate of BTV-1_26-S6_ or BTV-1_26-S7_ in C6/36 cells, although both viruses reach the same plateau level as BTV-1 by day 14 p.i.

Although the surface of BTV core-particles is composed entirely of VP7 (25), cores can still infect either BHK-21 or C6/36 cells with low efficiency, but show a much higher specific infectivity, which is comparable to that of intact virus particles, in KC cells, indicating that VP7 can mediate cell attachment and penetration in these *Culicoides* cells [14]. The RGD motif present in VP7 has been reported as essential for cell-binding/entry by viral cores [65]. Our results show that although VP7 of BTV-26 still contains the RGD motif, it is associated with a reduced rate of replication in KC cells. It is unclear if this reduction reflects a reduced efficiency in the initiation of infection, a delay in replication, or a reduced efficiency of virus assembly within the host cell cytoplasm. Comparison of VP7 from the two parental strains of BTV-1 and BTV-26, show only 19 aa differences (94.6% amino acid identity: Table 3). The significance of these differences in the reduced rate of replication of BTV-1_26-S7_ in KC cells is currently being investigated.

**Table 3:** Sequence identities between BTV-1 [RSArrrr/01] and BTV-26 [KUW2010/02].

Segment	% nucleotide identity	% amino acid identity
Seg-1 (VP1)	75.9	88.3
Seg-2 (VP2)	50.6	38.5
Seg-3 (VP3)	76.0	88.5
Seg-4 (VP4)	73.7	80.4
Seg-5 (NS1)	73.3	80.3
Seg-6 (VP5)	67.4	73.2
Seg-7 (VP7)	79.2	94.6
Seg-8 (NS2)	72.1	69.8
Seg-9 (VP6, NS4)	71.4	60.9 (VP6), 76.7 (NS4)
Seg-10 (NS3/3A)	79.2	85.6

Sequences for BTV-1 [RSArrrr/01] linked to Genbank accession numbers FJ969719 to FJ969728, and for BTV-26 [KUW2010/02] accession numbers JN255156 to JN255162 and HM590642 to HM590644.

Two mono-reassortants, BTV-1_26-S1_ and BTV-1_26-S3_ were unable to replicate in KC cells. Since both reassortants have functional cell entry proteins (VP2, VP5 and VP7) of BTV-1 and can infect BSR cells efficiently, it appears likely that they can also initiate infection in KC cells. However, during time course experiments, the viral RNA concentration gradually decreased over the 14 day incubation period, indicating that they did not replicate and their genomes were gradually being degraded. Interestingly, these two viruses showed quite different replication kinetics in C6/36 cells, which provides some clues to the mechanisms involved. BTV-1_26-S3_ showed no replication in C6/36 cells for the first 7 days, whereas BTV-1_26-S1_ replicated relatively well in these cells. This suggests that the phenotype of BTV-1_26-S3_ might not be specific to KC cells, but could be a result of the low temperature used for both insect cell lines compared to the BSR replication experiments. Since Seg-3 codes for the inner core protein, VP3, a protein essential for core assembly, it is possible that viral assembly is negatively affected at low temperature. In contrast, the phenotype of BTV-1_26-S1_, which has the BTV-26 polymerase, is more specific to KC cells. This is difficult to explain since VP1 functions within cores. One hypothesis is that this mono-reassortant is more susceptible to the antiviral pathways in *Culicoides* cells. Further investigations will be required to elucidate the reasons for the replication-negative phenotypes of these viruses in KC cells.

It was interesting that both BTV-1_26-S1_ and BTV-1_26-S3_ were more difficult to rescue than the other mono-reassortants, although these rescued viruses subsequently replicated normally in BSR cells, suggesting they had normal protein functions. Similarly, it has been reported that the rescue of BTV containing particular mutations or deletions in Seg-10 was delayed [54, 66]. These studies showed that despite the successful rescue of deletions in the Seg-10 ORF, RNA inserts from several genome segments were found in Seg-10 very quickly after virus rescue [66]. Although the reasons for the delayed rescue of BTV-1_26-S1_ and BTV-1_26-S3_ remain unknown, it is possible that there are changes on other segments which might be relevant, for example, the additional Seg-3 band that was observed upon rescue of BTV-1_26-S1_ (Fig 1) might be important. Full genome sequencing might reveal other changes that could help explain the difficulties in virus rescue.

The mono-reassortant containing NS2 from BTV-26 (BTV-1_26-S8_) replicated more slowly than BTV-1 [RSArrrr/01] in all three cell types, suggesting that the heterologous NS2 caused a general loss of fitness. This may result from a partial mismatch between NS2 of BTV-26 and the other viral components from BTV-1. NS2 of BTV-1 [RSArrrr/01] and BTV-26 have 107 amino acid changes, which could be responsible for such a mismatch. Another possibility is that interactions between BTV-26 NS2 and cellular components are less effective than with BTV-1 NS2. Substitution of Seg-8 in mono-reassortants between BTV-1 and BTV-8 also generated consistently smaller plaques than those of the two parental viruses [60].

The finding that the BTV-26 parental virus could not be rescued could be a result of its slightly slower replication in BSR cells compared to the BTV-1 parent (Fig 2). Alternatively, it might be a result of the cumulative negative effect of the conservative differences in some of the BTV-26 plasmid clones that together result in failure to rescue BTV-26.

Although BTV-26 and other BTV serotypes can replicate in C6/36 cells [13] the epidemiological significance is unclear, as mosquitos are not usually regarded as an important vector for BTV. Initial studies indicate that BTV-26 cannot infect adult *C*. *sonorensis*, although mosquito infection studies are still in progress. The failure of BTV-26 to replicate in KC cells, or infect *C*. *sonorensis* midges (unpublished), suggests that it may have lost the ability to be transmitted by adult *Culicoides*, although we cannot exclude the possibility that other *Culicoides* spp. (for example those indigenous to Kuwait) might act as vectors. However, BTV-26 appears to have an increased ability compared to other BTV strains, to transmit horizontally, via direct animal to animal contact [46, 47]. It is possible that proteins responsible for the inability of BTV-26 to replicate in KC cells (VP1, VP2, VP3 and VP7) are also associated with the apparent increase in efficiency of contact transmission.

In summary, a reverse genetics approach was used to generate reassortants between BTV-1 and BTV-26, to investigate their replication in BSR, KC and C6/36 cells. Although, most genome segments of BTV-26 could be rescued as mono-reassortants in a BTV-1 backbone, there is evidence of reassortment-groups that reflect interactions between the different components of the virus-particle (for example VP2, VP5 and VP7). The significance of sequence differences in the RNAs and proteins involved in these groupings, between different serotypes or topotype, still need to be determined.

The inability of BTV-26 to infect/replicate in KC (*C*. *sonorensis*) cells was shown to be associated with differences in Seg-1/VP1, Seg-2/VP2, Seg-3/VP3 and Seg-7/VP7. This suggests that although BTV cell-attachment / entry mechanisms (which involve VP2), are effective for BTV-26 in BSR cells and C6/36 cells, they are unable to mediate infection in *C*. *sonorensis* cells, suggesting the absence of an appropriate receptor. The mechanisms that restrict replication of BTV reassortants containing VP1, VP3 or VP7 of BTV-26 in KC cells, have not been determined. It is possible that some component or mechanism present in BSR cells is essential for BTV replication, but is missing in KC cells. Alternatively some component or mechanism present in KC cells may be triggered by or target these RNAs or proteins (such as an innate immune responses, or silencing) restricting replication.

The identification of individual genome segments/proteins that restrict the replication of BTV-26 in KC cells, or in adult *C*.*sonorensis* (a known BTV-vector species), shows that vector-competence can vary dramatically for different BTV strains within an individual *Culicoides* species. It is also possible that the transmission efficiency of an individual BTV strain varies between different *Culicoides* species. This could be better explored if cell lines and colonized insects, were available for other *Culicoides* species. Variations in their transmission efficiency could potentially explain why the spread of different BTV strains/serotypes is not uniform, with certain strains/serotypes apparently restricted to a limited geographic range (e.g. the spread of BTV-2, 4, 9 and 16 in southern Europe), while others are more widely distributed (e.g. BTV-1, 8, and 14 in northern Europe). Some BTV strains/serotypes may be better adapted to different episystems [67] containing different *Culicoides* species or populations, or like BTV-26 may be capable of horizontal transmission in the absence of vector insects. Further analyses of the interactions between viral RNAs/proteins and components of the host cell, will help to elucidate the mechanisms involved in vector transmission and competence, as well as the viral proteins and mechanisms that can enhance horizontal transmission of certain BTV strains.

## References

[1] Weaver SC. Evolutionary influences in arboviral disease. Current topics in microbiology and immunology. 2006;299:285–314. Epub 2006/03/30. . 16568903 10.1007/3-540-26397-7_10 PMC7120121

[2] Mertens PPC MS, Samuel A, Attoui H. Orbiviruses, Reoviridae In: Fauquet CM, Mayo MA, Maniloff J, Desselberger U, Ball LA, editors Virus Taxonomy Eighth Report of the International Committee on Taxonomy of Viruses London: Elsevier/Academic Press 2005:466–83.

[3] Alexander KA, MacLachlan NJ, Kat PW, House C, O'Brien SJ, Lerche NW, et al Evidence of natural bluetongue virus infection among African carnivores. The American journal of tropical medicine and hygiene. 1994;51(5):568–76. Epub 1994/11/01. . 7985748

[4] Meyer G, Lacroux C, Leger S, Top S, Goyeau K, Deplanche M, et al Lethal bluetongue virus serotype 1 infection in llamas. Emerging infectious diseases. 2009;15(4):608–10. Epub 2009/04/01. 10.3201/eid1504.081514 . 19331746

[5] Ruiz-Fons F, Reyes-Garcia AR, Alcaide V, Gortazar C. Spatial and temporal evolution of bluetongue virus in wild ruminants, Spain. Emerging infectious diseases. 2008;14(6):951–3. Epub 2008/05/30. 10.3201/eid1406.071586 18507912 PMC2600300

[6] Darpel KE, Batten CA, Veronesi E, Shaw AE, Anthony S, Bachanek-Bankowska K, et al Clinical signs and pathology shown by British sheep and cattle infected with bluetongue virus serotype 8 derived from the 2006 outbreak in northern Europe. The Veterinary record. 2007;161(8):253–61. . 17720961 10.1136/vr.161.8.253

[7] Howerth EW, Greene CE, Prestwood AK. Experimentally induced bluetongue virus infection in white-tailed deer: coagulation, clinical pathologic, and gross pathologic changes. American journal of veterinary research. 1988;49(11):1906–13. Epub 1988/11/01. . 2854709

[8] Wilson AJ, Mellor PS. Bluetongue in Europe: past, present and future. Philosophical transactions of the Royal Society of London Series B, Biological sciences. 2009;364(1530):2669–81. Epub 2009/08/19. 10.1098/rstb.2009.0091 19687037 PMC2865089

[9] Maclachlan NJ, Guthrie AJ. Re-emergence of bluetongue, African horse sickness, and other orbivirus diseases. Veterinary research. 2010;41(6):35 Epub 2010/02/20. 10.1051/vetres/2010007 20167199 PMC2826768

[10] Belhouchet M, Mohd Jaafar F, Tesh R, Grimes J, Maan S, Mertens PP, et al Complete sequence of Great Island virus and comparison with the T2 and outer-capsid proteins of Kemerovo, Lipovnik and Tribec viruses (genus Orbivirus, family Reoviridae). The Journal of general virology. 2010;91(Pt 12):2985–93. Epub 2010/08/27. 10.1099/vir.0.024760-0 . 20739272

[11] Stott JL, Osburn BI, Alexander L. Ornithodoros coriaceus (pajaroello tick) as a vector of bluetongue virus. Am J Vet Res. 1985;46(5):1197–9. Epub 1985/05/01. . 2988383

[12] Shaw AE, Veronesi E, Maurin G, Ftaich N, Guiguen F, Rixon F, et al Drosophila melanogaster as a model organism for bluetongue virus replication and tropism. Journal of virology. 2012;86(17):9015–24. 10.1128/JVI.00131-12 22674991 PMC3416142

[13] King BM, Alders MA. Morphology of bluetongue virus-infected Aedes albopictus (C6/36) cell culture. Prog Clin Biol Res. 1985;178:289–94. Epub 1985/01/01. . 2989866

[14] Mertens PP, Burroughs JN, Walton A, Wellby MP, Fu H, O'Hara RS, et al Enhanced infectivity of modified bluetongue virus particles for two insect cell lines and for two Culicoides vector species. Virology. 1996;217(2):582–93. . 8610450 10.1006/viro.1996.0153

[15] Mertens PP, Brown F, Sangar DV. Assignment of the genome segments of bluetongue virus type 1 to the proteins which they encode. Virology. 1984;135(1):207–17. . 6328750 10.1016/0042-6822(84)90131-4

[16] Roy P. Bluetongue virus genetics and genome structure. Virus research. 1989;13(3):179–206. Epub 1989/07/01. . 2549746 10.1016/0168-1702(89)90015-4

[17] Belhouchet M, Mohd Jaafar F, Firth AE, Grimes JM, Mertens PP, Attoui H. Detection of a fourth orbivirus non-structural protein. PloS one. 2011;6(10):e25697 10.1371/journal.pone.0025697 22022432 PMC3192121

[18] Ratinier M, Caporale M, Golder M, Franzoni G, Allan K, Nunes SF, et al Identification and characterization of a novel non-structural protein of bluetongue virus. PLoS pathogens. 2011;7(12):e1002477 Epub 2012/01/14. 10.1371/journal.ppat.1002477 22241985 PMC3248566

[19] Stewart M, Hardy A, Barry G, Pinto RM, Caporale M, Melzi E, et al Characterisation of a second open reading frame in genome segment 10 of bluetongue virus. The Journal of general virology. 2015 Epub 2015/08/21. 10.1099/jgv.0.000267 . 26290332 PMC4806581

[20] Maan S, Maan NS, Samuel AR, Rao S, Attoui H, Mertens PP. Analysis and phylogenetic comparisons of full-length VP2 genes of the 24 bluetongue virus serotypes. The Journal of general virology. 2007;88(Pt 2):621–30. 10.1099/vir.0.82456-0 . 17251581

[21] Maan S, Maan NS, Ross-smith N, Batten CA, Shaw AE, Anthony SJ, et al Sequence analysis of bluetongue virus serotype 8 from the Netherlands 2006 and comparison to other European strains. Virology. 2008;377(2):308–18. 10.1016/j.virol.2008.04.028 . 18570969

[22] Maan S, Maan NS, van Rijn PA, van Gennip RG, Sanders A, Wright IM, et al Full genome characterisation of bluetongue virus serotype 6 from the Netherlands 2008 and comparison to other field and vaccine strains. PloS one. 2010;5(4):e10323 10.1371/journal.pone.0010323 20428242 PMC2859060

[23] Huismans H, Erasmus BJ. Identification of the serotype-specific and group-specific antigens of bluetongue virus. The Onderstepoort journal of veterinary research. 1981;48(2):51–8. Epub 1981/06/01. . 6273773

[24] Roy P. Functional mapping of bluetongue virus proteins and their interactions with host proteins during virus replication. Cell biochemistry and biophysics. 2008;50(3):143–57. Epub 2008/02/27. 10.1007/s12013-008-9009-4 . 18299997

[25] Pritchard LI, Gould AR, Wilson WC, Thompson L, Mertens PP, Wade-Evans AM. Complete nucleotide sequence of RNA segment 3 of bluetongue virus serotype 2 (Ona-A). Phylogenetic analyses reveal the probable origin and relationship with other orbiviruses. Virus research. 1995;35(3):247–61. . 7785314 10.1016/0168-1702(94)00072-k

[26] Nomikou K, Dovas CI, Maan S, Anthony SJ, Samuel AR, Papanastassopoulou M, et al Evolution and phylogenetic analysis of full-length VP3 genes of Eastern Mediterranean bluetongue virus isolates. PloS one. 2009;4(7):e6437 10.1371/journal.pone.0006437 19649272 PMC2713410

[27] Gumm ID, Newman JF. The preparation of purified bluetongue virus group antigen for use as a diagnostic reagent. Archives of virology. 1982;72(1–2):83–93. Epub 1982/01/01. . 6285867 10.1007/BF01314453

[28] Attoui H, Mohd Jaafar F, Belhouchet M, Aldrovandi N, Tao S, Chen B, et al Yunnan orbivirus, a new orbivirus species isolated from Culex tritaeniorhynchus mosquitoes in China. The Journal of general virology. 2005;86(Pt 12):3409–17. Epub 2005/11/22. 10.1099/vir.0.81258-0 . 16298988

[29] Attoui H, Mendez-Lopez MR, Rao S, Hurtado-Alendes A, Lizaraso-Caparo F, Jaafar FM, et al Peruvian horse sickness virus and Yunnan orbivirus, isolated from vertebrates and mosquitoes in Peru and Australia. Virology. 2009;394(2):298–310. Epub 2009/09/22. 10.1016/j.virol.2009.08.032 . 19766284

[30] Grimes JM, Burroughs JN, Gouet P, Diprose JM, Malby R, Zientara S, et al The atomic structure of the bluetongue virus core. Nature. 1998;395(6701):470–8. 10.1038/26694 . 9774103

[31] Roy P. Bluetongue virus proteins. The Journal of general virology. 1992;73 (Pt 12):3051–64. Epub 1992/12/01. . 1335020 10.1099/0022-1317-73-12-3051

[32] Firth AE. Bioinformatic analysis suggests that the Orbivirus VP6 cistron encodes an overlapping gene. Virology journal. 2008;5:48 Epub 2008/05/21. 10.1186/1743-422x-5-48 18489030 PMC2373779

[33] Huismans H, Els HJ. Characterization of the tubules associated with the replication of three different orbiviruses. Virology. 1979;92(2):397–406. Epub 1979/01/30. . 218352 10.1016/0042-6822(79)90144-2

[34] Huismans H, van Dijk AA, Bauskin AR. In vitro phosphorylation and purification of a nonstructural protein of bluetongue virus with affinity for single-stranded RNA. Journal of virology. 1987;61(11):3589–95. Epub 1987/11/01. 2822964 10.1128/jvi.61.11.3589-3595.1987 PMC255959

[35] Huismans H, van Staden V, Fick WC, van Niekerk M, Meiring TL. A comparison of different orbivirus proteins that could affect virulence and pathogenesis. Veterinaria italiana. 2004;40(4):417–25. Epub 2004/10/01. . 20422564

[36] Quan M, van Vuuren M, Howell PG, Groenewald D, Guthrie AJ. Molecular epidemiology of the African horse sickness virus S10 gene. The Journal of general virology. 2008;89(Pt 5):1159–68. Epub 2008/04/19. 10.1099/vir.0.83502-0 . 18420793

[37] Gould AR. The complete nucleotide sequence of bluetongue virus serotype 1 RNA3 and a comparison with other geographic serotypes from Australia, South Africa and the United States of America, and with other orbivirus isolates. Virus research. 1987;7(2):169–83. Epub 1987/04/01. . 3035818 10.1016/0168-1702(87)90078-5

[38] Purse BV, Mellor PS, Rogers DJ, Samuel AR, Mertens PP, Baylis M. Climate change and the recent emergence of bluetongue in Europe. Nature reviews Microbiology. 2005;3(2):171–81. Epub 2005/02/03. 10.1038/nrmicro1090 . 15685226

[39] Purse BV, Brown HE, Harrup L, Mertens PP, Rogers DJ. Invasion of bluetongue and other orbivirus infections into Europe: the role of biological and climatic processes. Revue scientifique et technique (International Office of Epizootics). 2008;27(2):427–42. Epub 2008/09/30. . 18819670

[40] Hofmann MA, Renzullo S, Mader M, Chaignat V, Worwa G, Thuer B. Genetic characterization of toggenburg orbivirus, a new bluetongue virus, from goats, Switzerland. Emerging infectious diseases. 2008;14(12):1855–61. Epub 2008/12/03. 10.3201/eid1412.080818 19046507 PMC2634640

[41] Chaignat V, Schwermer H, Casati S, Planzer J, Worwa G, Vanzetti T, et al Occurrence and spatial distribution of Toggenburg Orbivirus in Switzerland. Small Ruminant Research. 2010;93(2–3):157–64. 10.1016/j.smallrumres.2010.05.016

[42] Chaignat V, Worwa G, Scherrer N, Hilbe M, Ehrensperger F, Batten C, et al Toggenburg Orbivirus, a new bluetongue virus: initial detection, first observations in field and experimental infection of goats and sheep. Veterinary microbiology. 2009;138(1–2):11–9. Epub 2009/03/11. 10.1016/j.vetmic.2009.02.003 . 19272719

[43] Maan S, Maan NS, Nomikou K, Batten C, Antony F, Belaganahalli MN, et al Novel bluetongue virus serotype from Kuwait. Emerging infectious diseases. 2011;17(5):886–9. 10.3201/eid1705.101742 21529403 PMC3321788

[44] Maan S, Maan NS, Nomikou K, Veronesi E, Bachanek-Bankowska K, Belaganahalli MN, et al Complete genome characterisation of a novel 26th bluetongue virus serotype from Kuwait. PloS one. 2011;6(10):e26147 10.1371/journal.pone.0026147 22031822 PMC3198726

[45] Jenckel M, Breard E, Schulz C, Sailleau C, Viarouge C, Hoffmann B, et al Complete coding genome sequence of putative novel bluetongue virus serotype 27. Genome announcements. 2015;3(2). Epub 2015/03/15. 10.1128/genomeA.00016-15 ; PubMed Central PMCID: PMCPmc4357740. 25767218 PMC4357740

[46] Batten CA, Henstock MR, Steedman HM, Waddington S, Edwards L, Oura CA. Bluetongue virus serotype 26: infection kinetics, pathogenesis and possible contact transmission in goats. Veterinary microbiology. 2013;162(1):62–7. Epub 2012/09/19. 10.1016/j.vetmic.2012.08.014 . 22986055

[47] Batten C, Darpel K, Henstock M, Fay P, Veronesi E, Gubbins S, et al Evidence for transmission of bluetongue virus serotype 26 through direct contact. PloS one. 2014;9(5):e96049 Epub 2014/05/07. 10.1371/journal.pone.0096049 24797910 PMC4010411

[48] Sato M, Maeda N, Yoshida H, Urade M, Saito S. Plaque formation of herpes virus hominis type 2 and rubella virus in variants isolated from the colonies of BHK21/WI-2 cells formed in soft agar. Archives of virology. 1977;53(3):269–73. Epub 1977/01/01. . 193466 10.1007/BF01314672

[49] Wechsler SJ, McHolland LE, Tabachnick WJ. Cell lines from Culicoides variipennis (Diptera: Ceratopogonidae) support replication of bluetongue virus. Journal of invertebrate pathology. 1989;54(3):385–93. Epub 1989/11/01. . 2553822 10.1016/0022-2011(89)90123-7

[50] Igarashi A. Isolation of a Singh's Aedes albopictus cell clone sensitive to Dengue and Chikungunya viruses. The Journal of general virology. 1978;40(3):531–44. Epub 1978/09/01. . 690610 10.1099/0022-1317-40-3-531

[51] Attoui H, Billoir F, Cantaloube JF, Biagini P, de Micco P, de Lamballerie X. Strategies for the sequence determination of viral dsRNA genomes. Journal of virological methods. 2000;89(1–2):147–58. Epub 2000/09/21. . 10996648 10.1016/s0166-0934(00)00212-3

[52] Maan S, Rao S, Maan NS, Anthony SJ, Attoui H, Samuel AR, et al Rapid cDNA synthesis and sequencing techniques for the genetic study of bluetongue and other dsRNA viruses. Journal of virological methods. 2007;143(2):132–9. . 17433453 10.1016/j.jviromet.2007.02.016

[53] Boyce M, Celma CC, Roy P. Development of reverse genetics systems for bluetongue virus: recovery of infectious virus from synthetic RNA transcripts. J Virol. 2008;82(17):8339–48. 10.1128/JVI.00808-08 18562540 PMC2519640

[54] van Gennip RG, van de Water SG, van Rijn PA. Bluetongue virus nonstructural protein NS3/NS3a is not essential for virus replication. PloS one. 2014;9(1):e85788 Epub 2014/01/28. 10.1371/journal.pone.0085788 ; PubMed Central PMCID: PMCPmc3896414. 24465709 PMC3896414

[55] Matsuo E, Roy P. Minimum requirements for bluetongue virus primary replication in vivo. Journal of virology. 2013;87(2):882–9. Epub 2012/11/02. 10.1128/jvi.02363-12 23115294 PMC3554043

[56] Karber G. Beitrag zur kollektiven behandlung pharmakologischer reihenversuche. Exp Pathol Pharmakol. 1931;162:480–3.

[57] Maan NS, Maan S, Belaganahalli M, Pullinger G, Montes AJ, Gasparini MR, et al A quantitative real-time reverse transcription PCR (qRT-PCR) assay to detect genome segment 9 of all 26 bluetongue virus serotypes. Journal of virological methods. 2015;213:118–26. Epub 2014/12/09. 10.1016/j.jviromet.2014.11.012 . 25486080

[58] Grimes JM, Jakana J, Ghosh M, Basak AK, Roy P, Chiu W, et al An atomic model of the outer layer of the bluetongue virus core derived from X-ray crystallography and electron cryomicroscopy. Structure. 1997;5(7):885–93. Epub 1997/07/15. . 9261080 10.1016/s0969-2126(97)00243-8

[59] Zhang X, Boyce M, Bhattacharya B, Zhang X, Schein S, Roy P, et al Bluetongue virus coat protein VP2 contains sialic acid-binding domains, and VP5 resembles enveloped virus fusion proteins. Proceedings of the National Academy of Sciences of the United States of America. 2010;107(14):6292–7. Epub 2010/03/25. 10.1073/pnas.0913403107 20332209 PMC2852009

[60] Shaw AE, Ratinier M, Nunes SF, Nomikou K, Caporale M, Golder M, et al Reassortment between two serologically unrelated bluetongue virus strains is flexible and can involve any genome segment. Journal of virology. 2013;87(1):543–57. 10.1128/JVI.02266-12 23097432 PMC3536370

[61] van Gennip RG, van de Water SG, Potgieter CA, Wright IM, Veldman D, van Rijn PA. Rescue of recent virulent and avirulent field strains of bluetongue virus by reverse genetics. PloS one. 2012;7(2):e30540 Epub 2012/03/01. 10.1371/journal.pone.0030540 ; PubMed Central PMCID: PMCPmc3281837. 22363444 PMC3281837

[62] van Gennip RG, van de Water SG, Maris-Veldhuis M, van Rijn PA. Bluetongue viruses based on modified-live vaccine serotype 6 with exchanged outer shell proteins confer full protection in sheep against virulent BTV8. PloS one. 2012;7(9):e44619 Epub 2012/10/11. 10.1371/journal.pone.0044619 ; PubMed Central PMCID: PMCPmc3458051. 23049753 PMC3458051

[63] Anbalagan S, Cooper E, Klumper P, Simonson R, Hause B. Whole genome analysis of epizootic haemorrhagic disease virus identified limited genome constellations and preferential reassortment. The Journal of general virology. 2013 Epub 2013/11/06. 10.1099/vir.0.059659-0 . 24189621

[64] Nunes SF, Hamers C, Ratinier M, Shaw A, Brunet S, Hudelet P, et al A synthetic biology approach for a vaccine platform against known and newly emerging serotypes of bluetongue virus. Journal of virology. 2014;88(21):12222–32. Epub 2014/08/22. 10.1128/jvi.02183-14 ; PubMed Central PMCID: PMCPmc4248921. 25142610 PMC4248921

[65] Tan BH, Nason E, Staeuber N, Jiang W, Monastryrskaya K, Roy P. RGD tripeptide of bluetongue virus VP7 protein is responsible for core attachment to Culicoides cells. Journal of virology. 2001;75(8):3937–47. Epub 2001/03/27. 10.1128/jvi.75.8.3937-3947.2001 11264382 PMC114884

[66] Feenstra F, van Gennip RG, van de Water SG, van Rijn PA. RNA elements in open reading frames of the bluetongue virus genome are essential for virus replication. PloS one. 2014;9(3):e92377 Epub 2014/03/25. 10.1371/journal.pone.0092377 ; PubMed Central PMCID: PMCPmc3962428. 24658296 PMC3962428

[67] Tabachnick WJ. Challenges in predicting climate and environmental effects on vector-borne disease episystems in a changing world. The Journal of experimental biology. 2010;213(6):946–54. Epub 2010/03/02. 10.1242/jeb.037564 . 20190119

